# Relationship between the computed tomography pattern and vaccination status in individuals with COVID-19

**DOI:** 10.1590/0100-3984.2025.0004

**Published:** 2025-07-17

**Authors:** Laís Rodrigues Bertoche, Waldinei Mercês Rodrigues, Caroline von Abel de Sousa, Vitor Maltoni Damasio, Hélder Jorge Andrade Gomes, Eduardo Vieira Ponte

**Affiliations:** 1 Department of Internal Medicine, Faculdade de Medicina de Jundiaí (FMJ), Jundiaí, SP, Brazil

**Keywords:** Tomografia computadorizada por raios X, Tórax, COVID-19, Vacinas, Tomography, X-ray computed, Thorax, COVID-19, Vaccines

## Abstract

**Objective:**

To evaluate the frequency of the typical computed tomography (CT) pattern in individuals with coronavirus 2019 (COVID-19), comparing those who were vaccinated with those who were unvaccinated.

**Materials and Methods:**

This was a retrospective study of the medical records of patients with clinical suspicion of COVID-19 between August 2021 and February 2022. The vaccination status was classified as absent/incomplete (0 or 1 dose) or complete (2 or more doses). The pattern seen on the first chest CT was defined as typical, atypical, indeterminate, or normal, the last three patterns being combined to form what was designated the non-typical group.

**Results:**

Binary logistic regression analysis showed that individuals with a complete vaccination status were less likely to present with the typical CT pattern than were those with an absent/incomplete vaccination status (adjusted OR = 0.19, 95% CI: 0.06-0.60).

**Conclusion:**

This information is important because it demonstrates that the frequency of the CT pattern considered typical of COVID-19 is currently lower than it was before the vaccines became available. Therefore, the typical CT pattern is no longer expected.

## Abstract (pt)

**Objetivo:**

Avaliar a frequência do padrão da tomografia computadorizada (TC) de indivíduos com COVID-19, comparando os vacinados com os não vacinados.

**Materiais e Métodos:**

Estudo retrospectivo em que foram selecionados prontuários de pacientes com suspeita clínica de COVID-19, entre agosto/2021 e fevereiro/2022. Os pacientes foram classificados, conforme a situação vacinal, em vacinação ausente/incompleta (0 ou 1 dose) ou vacinação completa (2 ou mais doses). A primeira TC de tórax do paciente foi classificada como típica, atípica, indeterminada ou normal, agrupando-se os padrões atípico, indeterminado ou normal em não típico.

**Resultados:**

A análise de regressão logística binária mostrou que indivíduos com vacinação completa tinham menor probabilidade de apresentar um padrão típico na TC de tórax em comparação com indivíduos com vacinação ausente/incompleta (OR ajustado: 0,19, IC 95%: 0,06-0,60).

**Conclusão:**

Esta informação é importante porque demonstra que, atualmente, a frequência de padrões tomográficos típicos é menor e, portanto, o padrão tomográfico típico não é mais esperado como foi visto previamente antes da vacinação.

## INTRODUCTION

Some individuals infected with severe acute respiratory syndrome coronavirus 2 (SARS-CoV-2) develop acute respiratory failure. During the coronavirus disease 2019 (COVID-19) pandemic, the Radiological Society of North America created a guideline that established the radiological pattern considered typical of COVID-19 and identified three other patterns to classify suspicious cases, designated the indeterminate, atypical, and normal patterns^**(^1^)**^. The characteristics on chest computed tomography (CT) considered typical of COVID-19 were multiple, bilateral subsegmental or lobular consolidations or bilateral ground-glass opacities^**(^2^,^3^)**^. In the period before the vaccines against COVID-19 became available, the CT pattern was important to differentiate COVID-19 from other causes of acute respiratory failure that could affect individuals infected with SARS-CoV-2, such as venous thromboembolism, decompensated heart failure, and bacterial pneumonia.

The development and administration of COVID-19 vaccines have changed the clinical and epidemiological scenario. The incidence of COVID-19 reduced rapidly; if diagnosed with the disease, vaccinated individuals had better clinical outcomes and less extensive lung involvement on chest CT^**(^4^-^8^)**^. However, little is known about the effect of the vaccine on the chest CT pattern in individuals with COVID-19. We hypothesized that the CT pattern considered typical of COVID-19 would be less common in vaccinated individuals than in those who were unvaccinated. Clarifying this issue is important because the absence of the typical CT pattern might not be sufficient to exclude the diagnosis of COVID-19 in vaccinated individuals who present hypoxemia during a SARS-CoV-2 infection. Therefore, the objective of this study was to determine whether, among individuals with COVID-19, the frequency of the typical CT pattern differed between vaccinated and unvaccinated individuals.

## MATERIALS AND METHODS

This was a retrospective study conducted in the city of Jundiaí, SP, Brazil, a municipality with 400,000 inhabitants. We reviewed the medical records of all individuals with clinical suspicion of COVID-19 who presented to the emergency room of a public hospital between August 2021 and February 2022. We selected the records of individuals ≥ 18 years of age with SARS-CoV-2 infection confirmed by reverse transcription-polymerase chain reaction (RT-PCR). Individuals in whom the CT image was of poor quality were excluded, as were those in whom the CT findings were consistent with an expansile process or fibrotic changes. The study was approved by the local institutional review board (Reference no. 5.711.929). Because the data were collected retrospectively, the requirement for informed consent was waived.

### Data retrieve

During the COVID-19 pandemic, the hospital protocol established in the city stated that individuals who presented to health care facilities with hypoxemia should undergo RT-PCR or an antigen test for SARS-CoV-2, as well as a chest CT scan, and be hospitalized. The electronic medical records of all such individuals were screened for this study. This study criteria for a diagnosis of COVID-19 were peripheral oxygen saturation below 94% on hospital admission and positivity for SARS-CoV-2 infection on one of the confirmatory tests. The electronic medical records included patient data collected systematically for epidemiological study by the Jundiaí Municipal Health Department: vaccination status; comorbidities (asthma, chronic obstructive pulmonary disease, congestive heart failure, hypertension, diabetes mellitus, and chronic renal failure); and smoking history. All of those data were retrieved for the purposes of this study.

### Vaccination status and analysis of chest CT

At the time of hospital admission, a health professional systematically reviewed the vaccination card of each individual to record the number of doses of COVID-19 vaccine received. This information was retrieved by researchers to classify the vaccination status of individuals included in the study as absent/incomplete (zero or one dose) or complete (two or more doses).

For each individual, the first chest CT scan was retrieved and evaluated by three radiologists who were blinded to the vaccination status. The images were classified by consensus as typical, atypical, indeterminate, or normal according to the Radiological Society of North America Expert Consensus Statement^**(^1^)**^. For statistical analysis purposes, we combined the atypical, indeterminate, and normal patterns into a group designated the non-typical group. Subsequently, we quantified the extent of lung involvement on CT. Each segment of each lung lobe was scored for the extent of involvement^**(^4^)**^: 1 point if 0-5%; 2 points if 5-25%; 3 points if 25-50%; 4 points if 50-75%; and 5 points if 75-100%. Those scores were summed to establish a final score, which was used in order to classify the overall extent of lung involvement as mild (≤ 8 points), moderate (9-15 points), or severe (> 15 points).

### Statistical analysis

To calculate the sample size, the alpha was set at 0.05 and the power was set at 80%. We estimated the minimum sample size to be 62 individuals in each group considering that a CT pattern typical of COVID-19 would be present in 70% of individuals with a complete vaccination status and in 90% of those with an absent/incomplete vaccination status^**(^9^)**^. We applied the chi-square and Mann-Whitney tests to compare categorical and continuous variables, respectively. We used binary logistic regression analysis to measure the association between vaccination status (independent variable) and the CT pattern (dependent variable). We adjusted the analyses for age, sex, comorbidities, smoking status, and the extent of lung involvement on chest CT because these covariates could affect the relationship between the dependent and independent variables^**(^10^)**^. The method of data entry into the regression model was the backward likelihood ratio approach. The level of significance required for a given variable to remain in the model was 0.10. We used the Hosmer-Lemeshow test to measure goodness-of-fit, as well as the tolerance test and variance inflation factor to measure collinearity. The data fitted the model well (Hosmer-Lemeshow test > 0.05), an we observed no collinearity (tolerance test > 0.10; variance inflation factor < 10).

## RESULTS

During the study period, 783 individuals presented with peripheral oxygen saturation less than 94%, and 274 of those individuals tested positive for SARS-CoV-2 infection by RT-PCR or antigen test. Of those 274 individuals, 58 were excluded: 27 because they did not undergo chest CT; 22 because their medical records did not contain information about their vaccination status; and nine for various other reasons. Therefore, the final sample comprised 216 individuals diagnosed with COVID-19.

Among the 216 individuals evaluated, 157 (73%) were fully vaccinated and 59 (27%) had an absent/incomplete vaccination status. Of those with a complete vaccination status, 84 (54%) were men, compared with 31 (52%) of those with an absent/incomplete vaccination status (*p* = 0.79). The median age in the two groups was 72 years (range, 64-79 years) and 58 years (range, 43-64 years), respectively (*p* < 0.01). Comorbidities were more common among individuals with a complete vaccination status than among those without, being identified in 131 (83%) and 36 (61%) of the individuals, respectively (*p* < 0.01).

**Figure 1:**
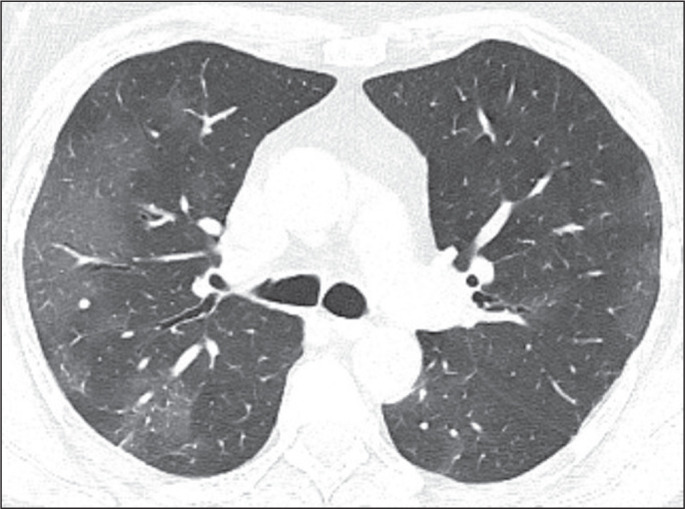
Typical CT findings. Axial CT showing ground-glass opacities with peripheral and bilateral distribution, classified as typical.

**Figure 2:**
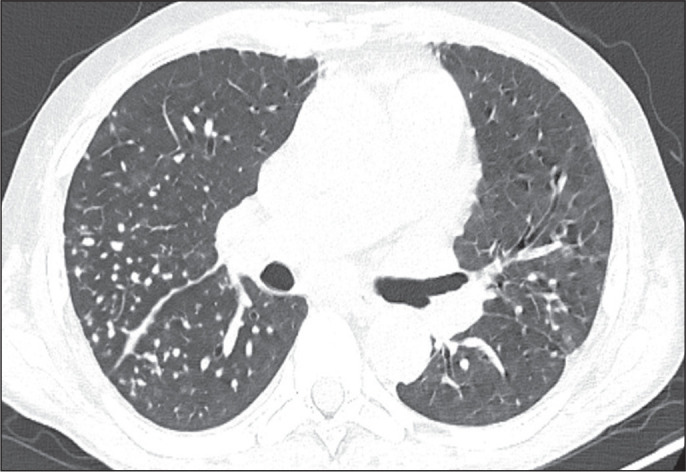
Atypical CT findings. Axial CT scan showing nodules with a centrilobular distribution.

**Figure 3:**
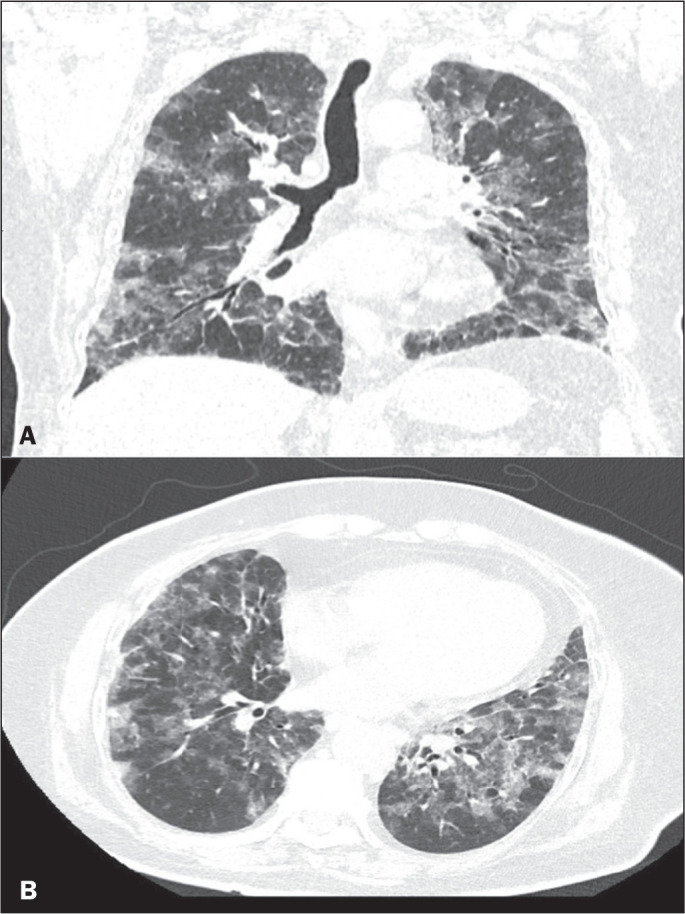
CT findings classified as severe in a 65-year-old unvaccinated female patient who complained of dyspnea, a runny nose, and myalgia. Coronal and axial CT scans (A and B, respectively) showing bilateral consolidations and opacities with ground-glass attenuation, with 20 points.

Typical CT pattern was observed in only 41% of the individuals with a complete vaccination status, compared with 83% of the individuals with an absent/incomplete vaccination status (*p* < 0.01) (Table 1). The binary logistic regression analysis showed that having a complete vaccination status reduced the chance of presenting the typical pattern on CT scan by 81% compared with having an absent/incomplete vaccination status (adjusted OR = 0.19, 95% CI: 0.06-0.60) (Table 2). Typical and atypical CT findings are illustrated in Figures 1 and 2, respectively. Ground-glass opacities and septal thickening were less common among individuals with a complete vaccination status than among those with an absent/incomplete vaccination status (adjusted OR = 0.20, 95% CI: 0.06-0.63 and adjusted OR = 0.41, 95% CI: 0.21-0.81, respectively), whereas there was no association between consolidations and vaccination status (adjusted OR = 0.71, 95% CI: 0.36-1.40). Additionally, individuals with a complete vaccination status were less prone to have moderate/severe lung involvement on chest CT (adjusted OR = 0.17, 95% CI: 0.07-0.41). Figure 3 illustrates CT findings classified as severe.

**Table 1:** Characteristics of patients with a confirmed diagnosis of COVID-19 who underwent chest CT, by vaccination status.

Characteristic	Vaccination status		*P*
	Absent/ incomplete (n = 59)	Complete (n = 157)	
Male, n (%)	31 (52)	84 (54)	0.79
Age (years), median (IQR)	58 (43-64)	72 (64-79)	< 0.01
Comorbidities, n (%)	36 (61)	131 (83)	< 0.01
Current or previous smoking, n (%)	12 (20)	47 (30)	0.16
Ground-glass opacities, n (%)	55 (93)	123 (78)	0.01
Consolidation, n (%)	37(63)	82 (52)	0.17
Septal thickening, n (%)	36 (61)	67 (43)	0.02
Severity on CT scan, n (%)			
Absent or mild	7(12)	74 (47)	< 0.01
Moderate or severe	52 (88)	83 (53)	
CT pattern, n (%)			
None	2(3)	16 (10)	< 0.01
Typical	49 (83)	65 (41)	
Atypical	1(2)	20 (13)	
Undetermined	7(12)	56 (36)	

**Table 2:** Binary logistic regression analyses of the associations between vaccination status and chest CT findings.

Absent/incomplete vs. complete vaccination	Crude OR (95% Cl)	Adjusted OR (95% Cl)
Ground-glass opacities^*^	0.26 (0.09-0.78)	0.20 (0.06-0.63)
Septal thickening^*^	0.45 (0.26-0.88)	0.41 (0.21-0.81)
Consolidations^*^	0.65 (0.35-1.20)	0.71 (0.36-1.40)
Typical CT pattern^^*^†^	0.14 (0.06-0.32)	0.19 (0.06-0.60)
Moderate-to-severe involvement on CT^‡^	0.15 (0.07-0.35)	0.17 (0.07-0.41)

* Adjusted for age, sex, comorbidities, smoking status, and extent of the involvement on CT.

† Typical vs. non-typical. The atypical, indeterminate, and normal patterns were combined into the non-typical group.

‡ Adjusted for age, sex, comorbidities, and smoking status.

## DISCUSSION

The results of this study allow us to conclude that individuals with a complete vaccination status are much less likely to present a typical CT pattern at the time of hospitalization due to COVID-19 compared to individuals with an absent/incomplete vaccination status. According to the upper limit of the confidence interval of the adjusted binary logistic regression, vaccinated individuals who presented to the emergency room with COVID-19 were 40% less likely to have a typical CT pattern than were those who were not vaccinated. This result was not biased by the lesser extent of lung involvement on chest CT in vaccinated individuals, nor by the difference in age and frequency of comorbidities between the groups, because the analysis was adjusted for those confounding variables. In the current scenario of ample access to the vaccine, these data indicate that health care professionals should suspect the diagnosis of COVID-19 in individuals infected with SARS-CoV-2 who present to the emergency department with hypoxemia even when the CT pattern is not typical of the disease.

Although our sample is representative of only the population of Brazil, a study conducted in France also observed a relationship between complete vaccination and a lower frequency of the typical CT pattern in individuals with COVID-19, suggesting that this association is independent of ethnic and epidemiological characteristics^**(^11^)**^. We are unaware of other studies exploring this topic. Despite the retrospective design of our study, we believe that the quality of the data was adequate because the variables extracted from the electronic medical records were registered objectively and systematically for epidemiological monitoring.

In Brazil, the government offered vaccines from various laboratories, and our conclusions are therefore not restricted to a specific type of vaccine. Although our sample size was small, it was sufficient to detect a statistically significant difference between the groups. In addition, the recruitment strategy allowed the sample to be representative of the population of the municipality of Jundiaí. Although individuals treated at private hospitals were not evaluated in this study, such individuals correspond to only 10% of the population in the city. In Jundiaí, the mean family income is above the Brazilian national average, the human development index is high, and the population is predominantly White. Therefore, our results may not be generalizable to populations living in low-income areas or to other ethnicities.

We conclude that, at the time of hospitalization for COVID-19, individuals with a complete vaccination status are less likely to present a typical CT pattern than are those with an absent/incomplete vaccination status. This information is important in clinical practice because it demonstrates that the absence of the typical CT pattern does not rule out the diagnosis of COVID-19 in previously vaccinated individuals who present hypoxemia during infection with SARS-CoV-2.

## References

[1] Simpson S, Kay FU, Abbara S (2020). Radiological Society of North America Expert Consensus Statement
on Reporting Chest CT Findings Related to COVID-19. Endorsed by the Society
of Thoracic Radiology, the American College of Radiology, and RSNA - Second
Publication. J Thorac Imaging.

[2] Huang C, Wang Y, Li X (2020). Clinical features of patients infected with 2019 novel
coronavirus in Wuhan, China. Lancet.

[3] Ebrahimzadeh S, Islam N, Dawit H (2022). Thoracic imaging tests for the diagnosis of
COVID-19. Cochrane Database Syst Rev.

[4] Yazdi NA, Ghadery AH, SeyedAlinaghi S (2021). Predictors of the chest CT score in COVID-19 patients: a
cross-sectional study. Virol J.

[5] Tabatabaei SMH, Talari H, Moghaddas F (2020). CT features and short-term prognosis of COVID-19 pneumonia: a
single-center study from Kashan, Iran. Radiol Cardiothorac Imaging.

[6] Baden LR, El Sahly HM, Essink B (2021). Efficacy and safety of the mRNA-1273 SARS-CoV-2
vaccine. N Engl J Med.

[7] World Health Organization Coronavirus disease (COVID-2019) pandemic.

[8] Mathieu E, Ritchie H, Ortiz-Ospina E (2021). A global database of COVID-19 vaccinations. Nat Hum Behav.

[9] Zhao W, Zhong Z, Xie X (2020). Relation between chest CT findings and clinical conditions of
coronavirus disease (COVID-19) pneumonia: a multicenter
study. AJR Am J Roentgenol.

[10] Possari RY, Andrade-Gomes HJ, Mello VC (2021). Association of coronary calcification with prognosis of Covid-19
patients without known heart disease. Braz J Med Biol Res.

[11] Crombé A, Bensid L, Seux M (2023). Impact of vaccination and the omicron variant on COVID-19-related
chest CT findings: a multicenter study. Radiology.

